# Extracellular vesicles in bacterial and fungal diseases – Pathogenesis to diagnostic biomarkers

**DOI:** 10.1080/21505594.2023.2180934

**Published:** 2023-03-06

**Authors:** Jnana A., Sadiya S. S., Satyamoorthy K., Murali T.S.

**Affiliations:** a Department of Biotechnology, Manipal School of Life Sciences, Manipal Academy of Higher Education, Manipal, India; b Department of Cell and Molecular Biology, Manipal School of Life Sciences, Manipal Academy of Higher Education, Manipal, India

**Keywords:** Biomarkers, extracellular vesicles, infection diagnosis, microbial pathogenesis

## Abstract

Intercellular communication among microbes plays an important role in disease exacerbation. Recent advances have described small vesicles, termed as “extracellular vesicles” (EVs), previously disregarded as “cellular dust” to be vital in the intracellular and intercellular communication in host-microbe interactions. These signals have been known to initiate host damage and transfer of a variety of cargo including proteins, lipid particles, DNA, mRNA, and miRNAs. Microbial EVs, referred to generally as “membrane vesicles” (MVs), play a key role in disease exacerbation suggesting their importance in pathogenicity. Host EVs help coordinate antimicrobial responses and prime the immune cells for pathogen attack. Hence EVs with their central role in microbe-host communication, may serve as important diagnostic biomarkers of microbial pathogenesis. In this review, we summarize current research regarding the roles of EVs as markers of microbial pathogenesis with specific focus on their interaction with host immune defence and their potential as diagnostic biomarkers in disease conditions.

## Introduction

Microbial infections often involve a complex interplay of host, environment, and the microbe. Most often, microbes can be found associated with the host whilst causing no apparent harm or providing any advantages to the host. However, when the host immunity is compromised, some of the “commensal” bacteria can turn into pathogens since the host immunity is weakened. Several efforts have been made to uncover genomic patterns delineating pathogens from commensals [1,2], with considerable attention towards factors that enhance the virulence of the pathogen. Some of the virulence factors that have received wide attention include toxin A and B produced by *Clostridium difficile* [3] and pathogenicity islands such as SPI-2 in *Salmonella* species [4]. While it is often presumed that virulence is a constant feature associated with pathogens, several researchers have shown that virulence can be easily attenuated and even enhanced for the same pathogenic microbe [5].

Therefore, it can be argued that a simplistic pathogen-centric approach which distinguishes a pathogen from a non-pathogenic microbe based merely on the presence or absence of few genetic elements such as virulence and resistance genes [6] might not fully unveil the complexity of host-pathogen interactions in microbial pathogenesis. Further, host immunity can play a major role in determining the disease severity; for instance, avirulent pathogens can cause severe symptoms in immunocompromised hosts; whereas if the host has a strong immunity, even virulent pathogens cause little to no symptoms [7]. For example, *Escherichia coli*, a ubiquitous commensal of human intestine, has been shown to mutate and acquire pathoadaptive traits such as increased survival and ability to escape macrophages in response to selective pressure from host’s innate immune cells [8]. Casadevall and Pirofski [9] proposed an elegant “damage response” framework to best determine the outcome of microbial pathogenesis considering both the pathogen and the host. This framework categorizes the outcomes of microbial interactions with the host as either a) commensalism, b) disease, or c) colonization based on the degree of damage suffered by the host as a function of time. It also considers both the microbe’s ability to cause disease as well as the immune responses mounted by the host to determine whether the interaction is favourable (equilibrium state) or not (disease) to the host. Therefore, considering that there is no true pathogen in the absence of a susceptible host, it might not be appropriate to search for “pathogenicity markers” only in the genomes of microbes. In addition, chronic infectious diseases are often associated with polymicrobial communities in a biofilm mode of growth as opposed to a single dominant pathogen [10].

The biofilm mode of microbial growth involves encasement of groups of microbes in a matrix of extrapolymeric substances [11]. The formation and maintenance of these biofilms require intricate networking in terms of microbial crosstalk and transfer of cellular cargo via vesicles to promote bacterial growth, provide the biofilm community with the required nutrients, immunity, armour (toxins and antimicrobial peptides) and coordinate microbe-host crosstalk in terms of immune modulation and evasion [12]. In response to pathogen attack, host immune cells release their own set of vesicular cargo to mount an effective defence and ensure an outcome (pathogen elimination) in its favour. These molecules include proteins such as mucins, complement system, interferon induced proteins, and protein complexes such as inflammasomes [13]. Coordination of these immune responses is, as in microbial biofilms, heavily reliant on communication signals and networks that are pivotal to coordinating an effective immune response [14]. Hence, rather than focussing on “pathogenicity markers” which is limited to the abilities of the microbe, one can look for molecules that are intricately involved in the inter and intracellular communication between the microbes and the host immune cells. One such candidate with immense potential in diagnosis and therapy is extracellular vesicles.

Extracellular vesicles (EVs) are released by cells across all kingdoms of life, prokaryotic and eukaryotic alike [15]. Largely disregarded as “cellular dust” earlier [16], EVs have gained increasing recognition as pivotal communication modules in host-microbe interactions, both between microbes (inter/intra species biofilms) and with the host immune cells [17]. These nano-sized, non-replicating, membranous structures have been suggested to play a crucial role in microbial quorum sensing by acting as vehicles for the diffusible signalling molecules such as acyl homoserine lactones [18]. EVs have been shown to aid in the transport of Pseudomonas quinolone signal (PQS) - a critical, hydrophobic quorum sensing molecule that regulates the transcription of several genes involved in virulence of *Pseudomonas aeruginosa* [19]. EVs have also been reported to be released more frequently by the pathogenic bacteria in comparison to their non-pathogenic strains [20]. EV cargo such as RNA has been shown to promote pathogenicity by various mechanisms including degradation/silencing of mRNA transcripts of host immune cells such as macrophages which lead to enhancement of microbial virulence [21,22]. EVs from host cells have been found to vary in composition among healthy and diseased individuals in response to bacteria [23,24]. EVs released from infected host cells have been found to be enriched in potentially pathogenic microbial peptides making them attractive candidates for diagnosis of latent infectious diseases. For example, researchers have reported the presence of *Mycobacterium tuberculosis* specific peptides in EVs from serum of patients with latent tuberculosis infection that were absent in controls [25].

Hence, while sequence-based biomarkers that distinguish a pathogen from a commensal are blurred by a myriad of factors that do not consider host immune response, EVs may act as reliable molecular signatures to distinguish a “true” pathogen from harmless commensals in accordance with the tenets of the “damage response framework” for assessment of microbial pathogenesis. The current review summarizes the knowledge available to date on the EVs (both from the microbe and host) with regards to their role in microbial pathogenicity and their potential to serve as relevant and effective diagnostic biomarkers.

## Extracellular vesicles in infectious diseases

### What’s in a name?

**Figure 1.**
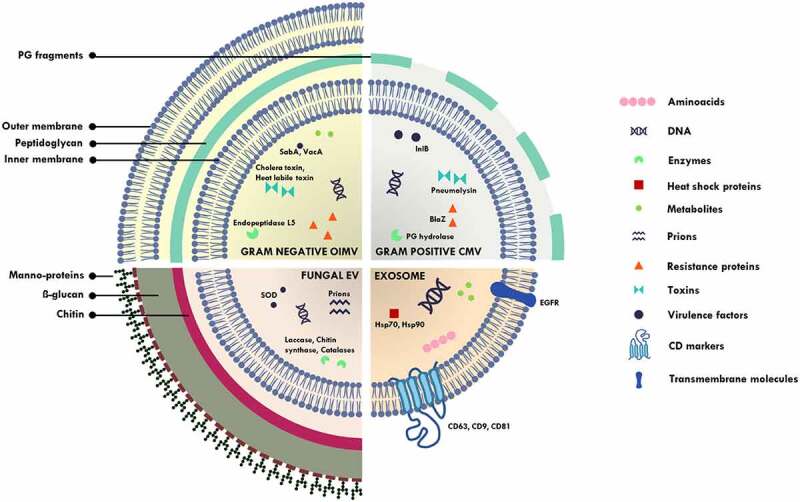
Schematic diagram illustrating outer inner membrane vesicles (OIMV) of Gram negative bacteria (containing cytoplasmic proteins in addition to the periplasmic space proteins in outer membrane vesicles), cytoplasmic membrane vesicles (CMV) of Gram positive bacteria, fungal extracellular vesicles and exosomes. SOD – superoxide dismutase, PG – peptidoglycan, SabA – sialic acid-binding adhesin, VacA – vacuolating cytotoxin, lnlB – *Listeria monocytogenes* protein, BlaZ – beta lactamase, Hsp – heat shock protein.

The observation of membranous structures being secreted outside the cell by mammalian cells was made as early as in the 1960s, most notably in the platelet studies by Peter Wolf wherein it was disregarded as “platelet dust” [16]. Several terms were used to describe such membranous structures by various research groups, though the term “exosomes” gained traction and became popular [26]. However, the observation of different membranous structures that varied in size and biogenesis resulted in an adoption of the term “extracellular vesicles.” This term is a hypernym that covers a range of nano-sized, non-replicating, membranous structures [27] that includes exosomes (40-160 nm, formed by inward budding of endosomes), ectosomes/microvesicles (50 nm-1 µm, formed by outward budding of plasma membranes) [28], apoptotic bodies (500-4000 nm, membrane blebbing of cells undergoing apoptosis) [29], outer-membrane vesicles (20-300 nm, vesicles released by Gram negative bacteria) [30] and membrane vesicles (20-100 nm, vesicles released by Gram positive bacteria, fungi, mycobacteria) [31]. In this review, we will restrict our discussion to exosomes (major EVs released by human host) and microbial vesicles (MVs – EVs released by microbes) (Figure 1).

### Where do they come from?

Biogenesis of exosomes and microbial vesicles has been extensively covered by several researchers in recent years [18,28,32–35]. The complexity and heterogeneity in EV cargo vary with the mechanism of biogenesis. Following is a brief summary of the different methods of vesicular release among microbes and the human host.

#### EVs released by microbes

The most widely studied membrane vesicles in bacteria are the outer membrane vesicles (OMV) observed primarily in Gram negative bacteria such as *P. aeruginosa* [36,37]. OMVs are composed of a phospholipid bilayer along with a thin layer of peptidoglycan enriched in periplasmic proteins such as alkaline phosphatase and envelope proteins [38]. In addition, the diderm nature of Gram negative bacteria makes way for outer-inner membrane vesicles (OIMV) which are enriched in both periplasmic and cytoplasmic proteins [30]. Biogenesis of OMVs is a regulated and possibly conserved mechanism across all species of Gram negative bacteria [33]. Though various mechanisms have been reported to induce vesiculation, the majority involve some form of envelope stress (loss of linkage between the outer membrane and the inner membrane). Vesiculation is also induced when exposed to external substances such as antimicrobials and hydrophobic molecules like hexadecane. A more “explosive” mode of OMV biogenesis has been noted in *P. aeruginosa* wherein rupture of the bacterial cell and the outer-membrane fragments for release of extracellular DNA (highly advantageous in the biofilm mode of life for the bacteria) leads to formation of membrane vesicles via revascularization of the resulting membrane fragments [35–39].

Release of vesicles by Gram positive bacteria and fungi was relatively less explored since it was thought that their thick cell walls would hinder this process; however, over the years, a significant amount of literature has been gathered on this aspect [31,37,40]. It is currently thought of as an essential process for survival as reported in Gram negative bacteria [41]. It has been suggested that in Gram positive bacteria, the peptidoglycan degrading enzymes might be involved in overcoming the “cell wall barrier” during vesiculation [42]. For example, endolysins have been shown to alter cell wall permeability, allowing for MV release in *Bacillus subtilis* [18,32].

Fungal MVs are bilayered vesicles enriched in enzymes that can degrade thick cell wall components, as has been observed in Gram positive bacteria [40]. They have been shown to have pivotal roles in induction of hyphal formation and regulation of the cell cycle [43]. Researchers have reported possible MV biogenesis pathways that involve the activity of endosomes and multivesicular bodies. Of note, members of the Endosomal Sorting Complex Required for Transport machinery (ESCRT) have been implicated in MV formation in *Candida albicans* and *Saccharomyces cerevisiae* [44–46]. Several other proteins such as SEC6, Golgi Reassembly And Stacking Protein (GRASP) and gene coding for phosphatidylserine decarboxylase have also been implicated in MV biogenesis and its regulation [46]. However, many of the mechanisms still remain unexplored.

#### EVs released by the host

Exosomes are spherical structures that can sometimes appear cup-shaped when imaged with transmission electron microscopy as a result of dehydration [47]. These are generally 60–120 nm in size with a phospholipid membrane rich in cholesterol, sphingomyelin, ceramide, and lipid rafts [28,48,49]. Based on cell size, exosomes are further divided as Exo – Large (Exo-L, 90-120 nm) and Exo – Small (Exo-S, 60-80 nm). Recently, a much smaller, non-membranous exosome has been characterized, defined as exomeres (35 nm). Biogenesis of exosomes has been covered extensively by recent literature [28]. In brief, exosomes are released by the inward budding of endosomes [50] and are characterized by the presence of specific markers such as CD9, CD63, CD81, Hsp70, flotillins, and transferrin receptor (Tsg101). The absence of glycophorin protein distinguishes it from ectosomes while the absence of CD31 and Annexin A5 distinguishes it from apoptotic bodies [51]. However, the conventional markers that distinguish exosomes from other EVs are unevenly represented among the three subpopulations of exosomes. CD9, CD63, and CD81 are universal markers for Exo-L/Exo-S population albeit with a cell and particle type specific expression, while FLOT1/2 are preferentially found in Exo-S population. Similarly, exomere populations are enriched in HSP90AB1 marker while HSP70 members are associated with Exo-L/Exo-S populations [52].

### Why are they important?

EVs have gained enormous traction in the medical field and in the understanding of infectious diseases. Membrane vesicles form the backbone of communication channels among the microbes enabling them to sense their surroundings and coordinate assault against the host to enhance pathogen mobility and establish infection [53]. They have been found to be crucial in establishing quorum sensing; a density dependent, chemical-based communication between microbes that is crucial for establishing multicellular scaffolds of biofilms. In Gram negative bacterium *Vibrio harveyi*, researchers have observed packaging of quorum sensing molecules in OMV facilitating its stability in the aqueous environments and enabling transfer over long distances [54]. Fungal MVs have been reported to carry a variety of proteins such as capsular polysaccharide glucuronoxylomannan (GXM) which offer a significant advantage during pathogenesis [55]. Falugi et al. [56] have reported Protein A, associated with *Staphylococcus aureus* cell walls, to be involved in immune evasion by blocking B cell responses during infection. Protein A has been reported to be an important cargo of *S. aureus* MVs [57]. A myriad of similar reports exist that highlight the involvement of MVs in biofilm formation, antibiotic protection, virulence, and immune modulation/evasion (Table 1).

**Table 1.** List of major extracellular vesicle cargo reported from bacteria and fungi.

FUNCTIONAL SIGNIFICANCE	EXTRACELLULAR VESICLE CARGO		
	Gram Negative Bacteria	Gram Positive Bacteria	Fungi
Biofilm formation	Long chain ketone CAI-1 (*V. harveyi*), Pseudomonas quinolone signal (PQS) (*P. aeruginosa*) [54]	Enterococcal surface protein (Esp), (*Enterococcus faecium*) [58], clumping factor adhesins such as ClfA, ClfB (*S. aureus*) [59]	Laccase (*Cryptococcus neoformans*) [31], beta hexosaminidase, chitinase (*C. albicans*) [60]
Drug susceptibility	β-lactamase (*P. aeruginosa*) [61], L1 metallo-β-lactamase and L2 serine-β-lactamase (*Stenotrophomonas maltophilia*) [62]	MsrR conferring methicillin resistance (*S. aureus*) [31], penicillin binding proteins (*S. aureus*) [59]	Putative glycanosyltransferase (Phr1) and putative endo-beta-D-glucosidase (Sun41) (*C. albicans*) [60]
Virulence	Pore­forming toxin ClyA (*Escherichia coli*) [31], cholera toxin (*Vibrio cholerae*) [54], CFTR inhibitory factor (*P. aeruginosa*) [63]	LLO (*Listeria monocytogenes*) [31], alpha toxin (Hla), cytolysins, leukocidin subunits such as LukS-PV, LukF-PV, LukE, LukD (*S. aureus*) [59]	Galactosaminogalactan (GAG) (*Aspergillus fumigatus*) [46], capsular polysaccharide glucuronoxylomannan (GXM) (*C. neoformans*) [55]
Immune modulation	Alkaline phosphatase, hemolytic phospholipase C (*P. aeruginosa*) [37], Cytotoxic necrotizing factor 1 toxin (*E. coli*) [64]	Protein A (*S. aureus*) [57], immune evasion factors such as Sbi, phenol-soluble modulins, catalase, SodA (*S. aureus*) [59]	Immunogenic GPI-anchored proteins, such as Phr1 (*C. albicans*) [46], GXM (*C. neoformans*) [55]

Exosomes hold enormous clinical potential in terms of therapy and diagnosis [52]. In the host, exosomes have been implicated in neuroprotection, cardiovascular and metabolic fitness, as well as in cancer progression [28]. Exosomes have also been described to have a protective role. Keller et al. [65] described these as “decoy exomes” that protect host cells by scavenging damaging proteins released by pathogens such as α toxins. Exosomes have also been reported as crucial communication mediators between the mother and foetus during pregnancy [66]. Exosomes released from placenta have been found to vary in various stages of pregnancy and show abnormal changes in pregnancy related complications such as preeclampsia, gestational diabetes, and pre-term birth [67]. Hence, they display great potential as a biomarker for diagnosis of pregnancy-related complications.

Of relevance to the current review is the importance of EVs in the various stages of infection. Exosomal contents and numbers have been demonstrated to vary in response to microbial infection and interaction with MVs released by microbial pathogens [68]. Briefly, the damage response process is initiated when the pathogen associated molecular patterns (PAMPs) of the microbes are recognised by pattern recognition receptors (PRRs) present on the host cell surface as well as in cytosolic compartments. Some of the cell surface PRRs include Toll like receptors (TLRs) such as TLR4 and TLR2 while intracellular PRRs include nucleotide binding oligomerization domain receptors (NLRs) such as NOD1 and NLRP6 [69–72]. These receptors enable recognition of invasive pathogens and upon pathogen challenge, initiate a signalling cascade to prevent pathogen colonization [70–73]. Generally, the host responds to the challenge with an initial inflammatory response which involves both innate and adaptive mechanisms aimed at destroying the pathogen and initiate clearance of debris, usually by stimulating cytokine secretion, antigen presentation, and inflammation [74,75]. The major immune cells involved in the first line of defence in microbial warfare are neutrophils and macrophages. Exosomes secreted by host macrophages following infection have been shown to be enriched in PAMPs such as lipopolysaccharides (LPS) and lipoproteins that are derived from the pathogen. These are capable of stimulating macrophages to release inflammatory mediators via Myd88 dependent signalling pathway, a crucial pathway governing inflammatory responses [75,76]. Exosomes are also involved in the adaptive immune response to microbial infection. For instance, exosomes derived from regulatory T cells have been reported to possess anti-inflammatory regulator CD3 which can modulate the growth of CD4+ T-cells [77]. Exosomes derived from activated T helper cells can interact with dendritic cells to initiate a cytotoxic T-cell response *in vivo* [78]. If the infection persists, then the host’s immune system triggers its cell signalling pathways to eliminate pathogens through programmed cell death [79,80].

Exosomes sometimes display a dichotomous behaviour wherein they can both harm and benefit the host [28]. For example, exosomes secreted by uninfected cells lying in the vicinity of infected cells can induce antiviral activity of IFN-α in the infected cell providing antiviral immunity and promoting host protection [81]. On the flip side, cytokine enriched exosomes from infected cells of host can also cause an escalation of the inflammatory reaction causing adverse effects in the host, especially in case of viral infections wherein the cytokines have a damaging role [82]. Hence there exist ample evidence from literature to support the exploration of EVs as potential biomarkers for detection and diagnosis of infectious diseases.

## Diagnostic potential of EVs

An ideal disease biomarker is one that is easily accessible for analysis (present in peripheral fluids such as blood or saliva to allow for non-invasive collection), easy to quantify with assays that are cost-effective, and is associated in a quantitative manner with the disease condition [83]. Some of the existing biomarkers for infection diagnosis include C-reactive protein (CRP) [84] and procalcitonin (PCT) for sepsis [85], calprotectin for acute respiratory infections [86], and several others [87]. While these biomarkers do meet the criteria for an ideal biomarker, they are not without disadvantages. For example, PCT surges in response to systemic infections, and hence, would not be an ideal marker of sepsis caused by localized infections [88]. Hence, there is still a need to find relevant biomarkers for the diagnosis and monitoring of infectious diseases. EVs with their pivotal roles in bacterial communication and host immunomodulatory properties can be potential biomarkers for diagnosis of infectious diseases [89–91].

Several qualities of EVs make them attractive biomarker candidates for infection diagnosis. The composition of EVs exchanged among pathogenic bacteria during host damage can serve as better biomarkers of pathogenic processes in comparison to biofluids which could be contaminated, thereby hindering rapid and specific diagnosis. In addition, unlike markers such as PCT that are relatively ineffective in detecting localized infections, EVs can be monitored for both localized and systemic infections [92].

Various methods exist for the isolation of EVs from easily accessible biological fluids such as blood, plasma, saliva and urine. These traditionally include ultracentrifugation, immunoaffinity-based characterization, size exclusion chromatography, polymer precipitation, and several others [93]. However, obtaining a good yield of EVs remains a challenge, especially in isolation of EVs from human sources such as blood and serum. Newer methods of EV isolation include microfluidic filtering (based on size of EVs) and contact-free sorting (based on size and density of EVs) [94]. Tulkens et al. [95] proposed a combination of techniques including ultrafiltration, size exclusion chromatography, and gradient centrifugation to allow for systematic separation of bacterial EVs from other molecules in human blood such as chylomicrons and high/low density lipoproteins. However, isolation of EVs remains a major bottleneck in utilising them as reliable biomarkers for routine use in clinical scenarios and requires significant innovation. Despite these, EVs have already shown incredible promise as biomarkers for the diagnosis of several diseases, especially breast cancer, ovarian cancer, prostate cancer, lung cancer and several others [93]. The potential of EVs to serve as diagnostic biomarkers has been explored even for infectious diseases caused by bacteria and fungi.

### EVs in bacterial infections

#### Candidate EV cargo of interest

With respect to infectious disease conditions, EV cargo such as adhesins, toxins, sRNAs [96], and immunomodulatory compounds derived from either the pathogen (MVs) or the infected host cells (exosomes) may serve as reliable candidates for diagnosis of infectious disease.

A fine example is the possibility of using MV cargo released from *P. aeruginosa* to diagnose cystic fibrosis. *P. aeruginosa* is known to release EVs with cargo containing Cif (cystic fibrosis transmembrane conductance regulator inhibitory factor) which can inhibit chlorine secretion pathways in lung epithelial cells causing a significant decrease in mucociliary clearance [97]. Virulence factors that significantly contribute to pathogenicity are attractive EV cargo that may serve as specific infectious disease biomarkers [9,98]. For example, granadaene, a pigmented and highly cytotoxic and haemolytic virulence protein produced by members of group B streptococci, has been reported to be majorly transported via MVs [99]. It has been shown to preferentially enhance survival of streptococci in host macrophages thereby perpetuating infection [100]. It also provides additional protection to the bacteria by inhibiting host innate immune responses such as release of antimicrobial reactive oxygen species (ROS) [101]. In another study, Gram negative *Serratia grimesii* OMVs were shown to be enriched with metalloprotease grimelysin, which is known to assist in eukaryotic host invasion [102].

#### Tuberculosis

**Figure 2.**
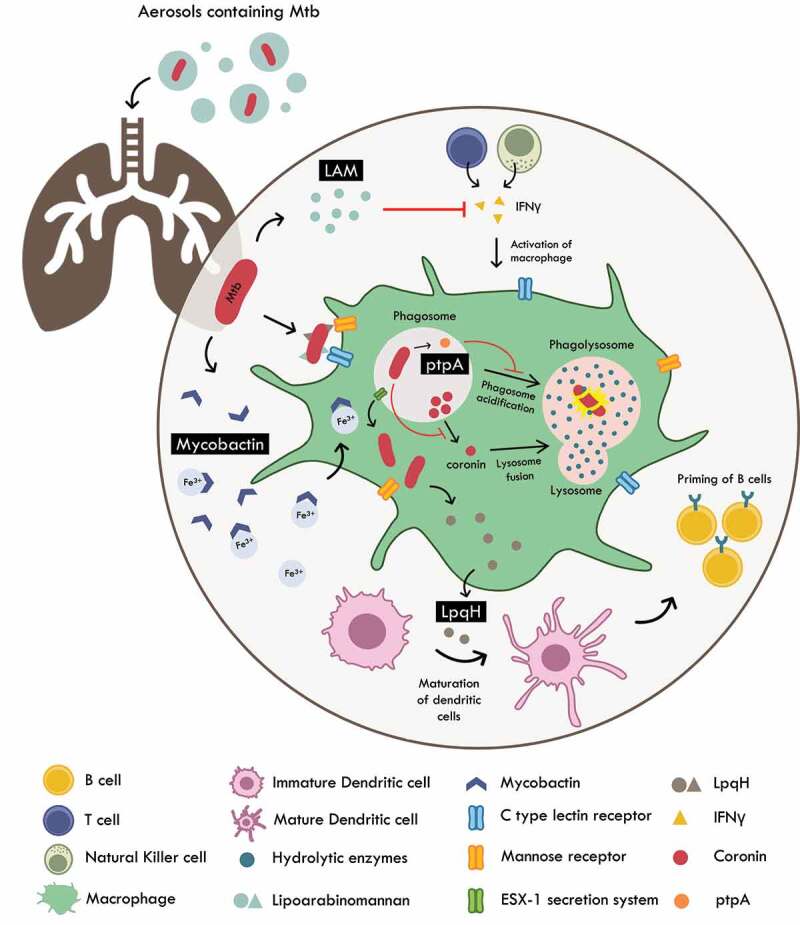
Host pathogen interactions of *M. tuberculosis*. Mtb is internalized by the macrophages via host receptors such as C – type lectin and mannose receptors that recognize LAM and LpqH respectively. Once internalized, the pathogen is subjected to intra-phagosomal processing. Mtb survives by producing molecules such as ptpA (that prevents phagosome acidification) and inhibition of secretion of coronin (important for lysosome – phagosome fusion). Mtb also induces T cell anergy and hampers the production of IFNγ by T cells and natural killer cells rendering them unable to activate the macrophage. LpqH released from lysis of Mtb are utilized by host to induce maturation of dendritic cells which then travel to lymph nodes to prime B cells for clearing of Mtb. Mtb is able to survive iron starvation by enhancing secretion of vesicles charged with mycobactin (siderophore). These key molecules can potentially serve as efficacious diagnostic biomarkers of TB.

Tuberculosis, a fatal and contagious infectious disease caused by the bacterium *Mycobacterium tuberculosis* (Mtb), has claimed 1.4 million lives worldwide and has the highest burden in India, followed by Indonesia and China [103]. In addition, around 2 billion people have latent Mtb and remain asymptomatic carriers of the disease [104]. Proper diagnosis and treatment with relevant antibiotics can effectively reduce the burden of TB as well as prevent the evolution and spread of multidrug resistant bacteria. Therefore, there is an imminent need for the development of novel biomarkers for diagnosis of TB [105]. Several groups of researchers have attempted to characterise and utilise MVs in TB for diagnosis. The primary molecules involved in defence against Mtb are macrophages (Figure 2). Mtb is engulfed by macrophages and subjected to intra-phagosomal processing with the aid of multiple hydrolases present within the macrophage. Mtb is able to overcome this by modulating the host macrophage environment to prevent its killing such as by inhibiting phagosome acidification and release of host factors such as coronin that promotes lysosome fusion [106]. Mtb also delays the involvement of adaptive immunity molecules by initiating production of anti-inflammatory molecules [107]. Sometimes, during host-pathogen interaction, Mtb gets retained in its latent form, where macrophages within the host serve as a reservoir of *Mycobacterium* in the form of a tuberculous granuloma. These granuloma macrophages possess necrotic potential and eventually release Mtb, by means of Esx-1 secretion system, into the lung interstitium to allow for further Mtb replication and spread into the neighbouring cells [108–110].

It is now well established that there is a significantly enhanced EV secretion during *Mycobacterium* infection. It is therefore possible that the immunomodulation of Mtb is coordinated via EVs which can be intercepted and assessed for molecules that can act as reliable biomarkers of the disease. One of the candidate molecules include Lpqh, a 19 kDa lipoprotein found in the cell wall of Mtb, that relays immunological information to other cells thereby regulating the host immune response in the pathogen’s favour [75,111]. This lipoprotein is known to be transported via exosomes secreted from *M. tuberculosis*-infected macrophages. Researchers have reported its application as an efficacious plasma biomarker to distinguish between paratuberculosis and tuberculosis infection in cows [112]. Alternatively, LpqH is also a potent TLR2 agonist that can influence T cell signalling and induce maturation of dendritic cells. The mature dendritic cells are then transported to lymph nodes wherein they can prime T cells in order to aid the host in pathogen defence [113]. Another molecule of diagnostic potential can be PtpA, a tyrosine phosphatase found in Mtb. PtpA is a powerful immunomodulatory molecule that aids in inhibition of phagosome acidification, effectively impairing the host macrophage’s ability to lyse Mtb [114]. PtpA has been reported to be transported by EVs, thus allowing for possible therapeutic monitoring of TB progression. Iron starvation is a common problem faced by pathogens during infection. Mtb is able to overcome this by enhancing secretion of siderophores such as mycobactin. This enhanced production of mycobactin has been correlated with increased vesicular packaging and transport via EVs [115], making them an attractive biomarker for non-invasive monitoring of TB progression [116].

There are considerable gaps in the knowledge regarding asymptomatic carriers of Mtb. It is not yet understood how some individuals experience active TB while several do not display any TB phenotype [117]. The difference is likely because in some hosts, Mtb is able to effectively escape immune detection and deviously modulate the host immune responses to allow for its replication and survival [118]. One of the molecules involved in down regulation of adaptive immune molecules such as CD4+ T cells resulting in T cell anergy is lipoarabinomannan (LAM). Production of LAM is one of several mechanisms employed by Mtb to evade immune recognition [119]. LAM has been shown to be transported via exosomes in an Mtb infection mice model. However, LAM alone does not fulfil the criteria to be a specific biomarker of latent TB since LAM has been described as a diagnostic marker for both active and latent TB [120]. Intriguingly, in an analysis of the components of MVs released by pathogenic *Mycobacterium bovis*, Bacillus Calmette-Guerin (BCG), Mtb, and non-pathogenic *Mycobacterium smegmatis*, MVs released by pathogens were alone found to be enriched in lipoprotein innate immune receptor agonists such as LpqH. These TLR2 agonists have been shown to induce production of immunosuppressive cytokines and CD4+ T cells [121]. An additional powerful immunomodulatory cargo transported by EVs are miRNA [122]. Mirzaei et al. [105] have explored exosomal miRNAs as a diagnostic and therapeutic biomarker for Mtb infection. Potential miRNA candidates include miR155, miR146a, miR132, and few others. However, their specific enrichment in exosomes needs further investigation. Lyu et al. [123] have also reported distinct exosomal miRNA profiles among healthy individuals, individuals with latent TB, and with active TB. These findings suggest that Mtb EVs (MVs and exosomes) play a crucial role in the pathogenesis of TB and is worth exploring as a diagnostic biomarker.

Another feature of a good biomarker is ease of accessibility for detection and analysis. Standard methods for diagnosis of TB include culture-dependent methods such as sputum smear microscopy to detect the bacilli and microscopic observation of multidrug resistant Mtb [124]. However, these methods are time consuming, require skilled practitioners and vary widely in terms of sensitivity and specificity [125]. Though, a few recent molecular methods including line probe assay and Xpert® MTB/RIF (*M. tuberculosis*/Rifampicin) assay can overcome these limitations, these are not cost-effective and are limited by the detection variabilities introduced by reported mutations in Mtb genome [126]. In addition, a successful microbial pathogenesis cannot be predicted merely by the genomic determinants of the microbe alone [9]. Therefore, EVs which can represent both microbial and host components of microbial pathogenesis may prove to be valuable and novel biomarkers for effective TB diagnosis. EVs can be obtained non-invasively from fluid samples such as urine and has been shown to be specifically enriched in immunomodulatory molecules that cause extensive host damage over the course of TB [107]. Dahiya et al. [127] explored a simple workflow for detection of Mtb from urinary EVs using an indirect immune-polymerase chain reaction with a limit of detection of 1fg/ml for the active TB markers lipoarabinomannan and culture filtrate protein-10. Mehaffy et al. [25] developed a detection technique based on multiple reaction monitoring mass spectrometry and were able to detect Mtb peptides even in EVs from serum of individuals with latent TB.

#### Pneumonia

Pneumonia is an infection of the lower respiratory tract [128] with higher mortality rates in children and subjects especially from lower, middle-income countries [129]. Causal agents of pneumonia include bacteria such as *Streptococcus pneumoniae* and *Klebsiella pneumoniae* [130]. Of these, *S. pneumoniae* is the leading causative agent of bacterial pneumonia globally [131]. MV production has been found to be a major virulence factor responsible for extensive damage of host tissue and manipulation of host immune responses [132]. These pathogen-derived vesicles are generally enriched in lipoproteins and have been found to ferry cytotoxic proteins such as pneumolysin which induce proinflammatory cytokine responses in the dendritic and lung epithelial cells (major host immune cells activated in pneumococcal infections). MVs secreted by *K. pneumoniae* consist of many virulence factors including adhesins, LPS and peptidoglycans [20]. LPS present within these MVs are essential in immune stimulation as they are known to induce thymus independent humoral immunity [133]. In addition to this, PAMPs such as adhesins, peptidoglycans, and outer membrane porins can act as ligands in interaction with host cells thereby promoting pro-inflammatory responses [134,135] by inducing expression of IL-1β and IL-8. These molecules function as mediators of local inflammation by recruiting neutrophils and monocytes at the site of inflammation leading to a strong immune response. However, whether this response causes clearance of infection or enhances the virulence is not clear [136]. Additionally, pneumococcal MVs evade complement mediated killing by binding to complement factors, hindering the interaction of bacteria with complement receptors on host cells [137]. *S. pneumoniae* has also been reported to release MVs enriched in endonucleases in response to neutrophil extracellular traps released by host neutrophils to degrade the bacterium [138]. Host cells also release exosomes in response to a pneumococcal infection. These were found to be enriched in miRNAs and cytokines and were primarily released by lung epithelial cells or infected macrophages. The unique signature of EVs from pneumonia patients highlight their potential as excellent diagnostic biomarkers [139–141].

The standard methods for diagnosis of pneumonia include culture-based methods involving microscopy, urinary antigen tests and biochemical tests for detection of respiratory pathogens and serology tests for detection of antibodies in blood and antigens in urine cultures. Molecular methods are routinely employed to decipher the microbe involved in the pathogenesis [142]. However, diagnosis of pneumonia remains a major diagnostic challenge riddled with misdiagnosis and false negatives [143]. Since, existing biomarker-based diagnostic methods such as X-ray, PCR, mass spectrometry, and immunoassay suffer from poor sensitivity, specificity and in some cases limited by the number of pathogens that can be detected; novel antimicrobial peptides have been explored for their diagnostic potential using *in silico* methods [144]. However, the interaction of these antimicrobial peptides with pathogen receptors needs further validation. Biomarker potential of small EVs in inflammatory airway diseases such as pneumonia has been extensively reviewed elsewhere [145]. As in TB, RNA cargo of EV has been extensively explored as an attractive candidate for specific diagnosis of pneumonia. Huang et al. [146] have identified potential miRNA biomarkers such as the ratio of expression of miR-450a-5p with that of miR-103a-3p from serum exosomes obtained from children with pneumonia as a consequence of adenovirus infection. Sun et al. [147] have reported mir175p and mir143A-5P to be enriched in exosomes isolated from bronchoalveolar lavage fluid of pneumonia patients. These exosomal miRNA signatures were exclusive to exosomes of patients with pneumonia strengthening their validity as useful and specific biomarkers for diagnosis of pneumonia. In addition, pneumonia is often misdiagnosed with other inflammatory respiratory diseases such as chronic obstructive pulmonary disease (COPD). Existing biomarkers such as procalcitonin (PCT) lack specificity and cannot distinguish pneumonia from COPD [148]. Hsu et al. [149] reported the possibility of using CRP in combination with sphingosine-1-phosphate to allow for demarcation of COPD and pneumonia. Jung et al. [150] identified surface proteins on plasma extracellular vesicles namely CD16, CD28, CD45 and TNF-R-II allowing for clear delineation of pneumonia from COPD. Since these EVs can be easily obtained from blood plasma, it presents itself as a potential biomarker for distinguishing pneumonia from COPD. Furthermore, considering their increased stability in body fluids and critical roles in immune modulation and toxin delivery, EVs hold enormous diagnostic potential as non-invasive and rapid disease indicators. However, there is an urgent need for accurate quantification and identification of EVs, a major bottleneck in widespread adoption of EVs for diagnosis [151].

### Fungal infections

Fungi are heterotrophic eukaryotes implicated in deadly infectious diseases such as invasive candidiasis and cryptococcosis whose mortality rates are similar to that of tuberculosis [31]. Fungi are highly proficient in tuning to their environment and can establish several relationships with their host organism ranging from commensal, symbiotic to pathogenesis [152]. Microbe-host relationships are heavily intertwined in fungal-human interactions since fungi have evolved with their hosts for generations leading to possible complex host immune signalling cascades and fungal specific host immune evasion mechanisms [153].

The existence of fungal MVs came into light in the last 15 years with the major focus on *C. neoformans* and since then there have been several reports on MVs from other fungi such as *S. cerevisiae, C. albicans, Histoplasma capsulatum, Pichia fermentans,* and *Cryptococcus gattii* [40,154–156]. *C. neoformans* is a major fungal pathogen causing cryptococcosis in immunocompromised individuals [56]. EVs of *C. neoformans* contain virulence factors such as polysaccharide glucuronoxylomannan which helps protect the fungus from phagocytosis and inhibits migration of leukocytes [157]. In addition to this, the EVs also carry glucosylceramide, acid phosphatase, laccase, urease, and several antioxidant proteins such as superoxide dismutase, thioredoxin dismutase, thioredoxin, and catalase A. Rodrigues et al. [158] showed that serum obtained from patients infected with *C. neoformans* had specific antibodies against proteins in the MVs secreted by the pathogen. Due to their foreign nature, MVs are capable of activating macrophages leading to increased production of TNF-α and other antimicrobial compounds with potential to restrict the fungal infection [159]. Other studies indicate the involvement of MVs in promoting fungal virulence. A study conducted on *C. neoformans* showed that *sec6* (involved in trafficking of exocytic vesicles and docking with plasma membrane for fusion) knockouts exhibited decreased virulence *in vivo* indicating MVs to be pivotal to fungal virulence [160]. A complete knockdown of this gene resulted in inhibition of MV production and blocked the transmission of a major virulence factor laccase [161], an enzyme required for the synthesis of melanin which contributes to fungal virulence by blocking the phagocytic activity of macrophages [162]. Researchers have also observed MVs secreted from *C. neoformans* to be internalized by macrophages. These macrophages are then trafficked to cryptococci residing in phagosome, thereby resulting in increased proliferation [163]. Several other fungal pathogens also facilitate delivery of different effector molecules in a similar fashion [154,156,164]. As in bacterial MVs, fungal MVs have been shown to carry powerful immunomodulatory cargo and are also attractive candidates as vaccines [165]. Interestingly however, *C. albicans* have been shown to induce host immune system into releasing exosomes enriched in TGF-β1 leading to development of immune tolerance and lowered host immune response, favouring survival of the pathogen [166].

Current methods of diagnosis of fungal infections involve culturing, microscopy, cytology [167], histopathology [168], and serology tests including detection of biomarkers such as β-D-glucan or *Aspergillus* galactomannan [169]. While these tests are of routine use in the clinical setting, they cannot conclusively diagnose invasive fungal diseases in a timely manner. Culture-independent tests or nucleic acid amplification tests involving amplification of barcodes such as internal transcribed spacer regions and next generation sequencing of multicopy genomic locus targets such as 18S rRNA and 5.8S rRNA enable rapid diagnostics [170]. Vaz et al. [171] developed a mass spectrometry-based diagnosis of invasive candidiasis while Koo et al. [172] reported a novel “volatile diagnostics” procedure wherein invasive aspergillosis was diagnosed by the presence of sesquiterpene metabolites in the breath of the patients. However, majority of the molecular tests lack validity, exhibit cross reactivity, are limited to detection of a few species, and are generally applied only to pure cultures [169]. Exploration of fungal EVs as diagnostic biomarkers is still at its infancy. However, a few research groups have explored the possibility and shortlisted few EV molecules that help in delineating pathogens from non-pathogens [173]. We propose that these EV molecules can be further probed for their effectiveness as a diagnostic biomarker. For example, Martinez-Lopez et al. [174] described a 20S proteasome complex exclusively in hyphal EVs of *C. albicans*. Since the yeast to hyphae transition is a major process that enhances fungal virulence, detection of these complexes can serve as early diagnostic biomarkers of invasive candidiasis. An extensive omics approach, as detailed by Zamith-Miranda et al. [175] can help enhance our knowledge in the area of fungal MV biology, biogenesis, composition, and immunomodulation capabilities aiding in discovery of new molecules with potential as diagnostic biomarkers.

## Conclusion

A diagnostic infection biomarker is ideally a molecule that can enable the diagnosis of an infectious disease caused by a microbial pathogen. However, pathogenicity is not a well-defined feature that can be represented by genomic/proteomic or biochemical signatures alone, since, commensal microbes can become pathogenic if an opportunity is presented by the host and the environment. Hence, host damage is an important tenet in the manifestation of an infection. Extracellular vesicles that mediate infection outcomes by enabling microbe-host crosstalk presents itself as an attractive vehicle in the diagnosis of infectious diseases. Their small size, capability to shuttle immunomodulatory cargo and toxic proteins, insolubility in biological fluids, and central role in host-microbe interactions make them an ideal biomarker that can be profiled non-invasively. However, it is important to note that EVs are released by both pathogen and non-pathogens alike. Hence, while EVs may offer sensitivity, their specificity needs to be further investigated to exclusively delineate pathogenic components. There also exist some minor bottlenecks in terms of EV collection, sizing, analysis and interpretation in the clinical scenario. Researchers have also reported changes in the EV cargo with time, hindering its reliability to spell out a single disease state. Therefore, it is crucial to further study the temporal variability of EV cargo with progression of disease and the subsequent changes in its cell surface markers. EV diagnosis and immunomodulatory potential also needs to be validated with *in vivo* studies so as to enable translation into clinical settings. Improvements in EV collection, sizing, and interpretation will vastly improve its access to hospital settings and get us one step closer to utilizing EVs as diagnostic biomarkers. The rise of multi drug resistant microbes and arrival of post antibiotic era highlights the need for novel approaches to curb infectious diseases. Further research and technological advancements in the field of extracellular vesicles can offer us with crucial weapons in our battle against the infectious pathogens.

## References

[1] Biek R, Pybus OG, Lloyd-Smith JO, et al. Measurably evolving pathogens in the genomic era. Trends Ecol Evol. 2015;30(6):306–16. 25887947 10.1016/j.tree.2015.03.009 PMC4457702

[2] Moran NA. Microbial minimalism. Cell. 2002;108(5):583–586. 11893328 10.1016/s0092-8674(02)00665-7

[3] Monot M, Eckert C, Lemire A, et al. *Clostridium difficile*: new insights into the evolution of the pathogenicity locus. Sci Rep. 2015;5(1):15023. 26446480 10.1038/srep15023 PMC4597214

[4] Lebeer S, Vanderleyden J, Keersmaecker SCJD. Host interactions of probiotic bacterial surface molecules: comparison with commensals and pathogens. Nature Rev Microbiol. 2010;8(3):171–184. 20157338 10.1038/nrmicro2297

[5] Pirofski L, Casadevall A. Q and a what is a pathogen? a question that begs the point. BMC Biol. 2012;10(1):6–6. 10.1186/1741-7007-10-6 PMC3269390 22293325

[6] Pallen MJ, Wren BW. Bacterial pathogenomics. Nature. 2007;449(7164):835–842. 17943120 10.1038/nature06248

[7] Casadevall A, Pirofski L. Host‐pathogen interactions: the attributes of virulence. J Infect Dis. 2001;184(3):337–344. 11443560 10.1086/322044

[8] Proença JT, Barral DC, Gordo I. Commensal-to-pathogen transition: one-single transposon insertion results in two pathoadaptive traits in *Escherichia coli* -macrophage interaction. Sci Rep. 2017;7(1):4504. 28674418 10.1038/s41598-017-04081-1 PMC5495878

[9] Casadevall A, Pirofski L. The damage-response framework of microbial pathogenesis. Nature Rev Microbiol. 2003;1(1):17–24. 15040176 10.1038/nrmicro732 PMC7097162

[10] Peters BM, Jabra-Rizk MA, O’may GA, et al. Polymicrobial interactions: impact on pathogenesis and human disease. Clin Microbiol Rev. 2012;25(1):193–213. 22232376 10.1128/CMR.00013-11 PMC3255964

[11] Costerton JW, Cheng KJ, Geesey GG, et al. Bacterial biofilms in nature and disease. Annu Rev Microbiol. 1987;41(1):435–464. 3318676 10.1146/annurev.mi.41.100187.002251

[12] Zaborowska M, Taulé Flores C, Vazirisani F, et al. Extracellular vesicles influence the growth and adhesion of *Staphylococcus epidermidis* under antimicrobial selective pressure. Front Microbiol. 2020;11:1132. 32714283 10.3389/fmicb.2020.01132 PMC7346684

[13] Medzhitov R. Recognition of microorganisms and activation of the immune response. Nature. 2007;449(7164):819–826. 17943118 10.1038/nature06246

[14] Xie J, Tato CM, Davis MM. How the immune system talks to itself: the varied role of synapses. Immunol Rev. 2013;251(1):65–79. 23278741 10.1111/imr.12017 PMC3645447

[15] Deatherage BL, Cookson BT. Membrane vesicle release in bacteria, eukaryotes, and archaea: a conserved yet underappreciated aspect of microbial life. Infect Immun. 2012;80(6):1948–1957. 22409932 10.1128/IAI.06014-11 PMC3370574

[16] Wolf P. The nature and significance of platelet products in human plasma. Br J Haematol. 1967;13(3):269–288. 6025241 10.1111/j.1365-2141.1967.tb08741.x

[17] Schorey JS, Cheng Y, Singh PP, et al. Exosomes and other extracellular vesicles in host–pathogen interactions. EMBO Rep. 2015;16(1):24–43. 25488940 10.15252/embr.201439363 PMC4304727

[18] Toyofuku M, Morinaga K, Hashimoto Y, et al. Membrane vesicle-mediated bacterial communication. Isme J. 2017;11(6):1504–1509. 28282039 10.1038/ismej.2017.13 PMC5437348

[19] Mashburn LM, Whiteley M. Membrane vesicles traffic signals and facilitate group activities in a prokaryote. Nature. 2005;437(7057):422–425. 16163359 10.1038/nature03925

[20] Kuehn MJ, Kesty NC. Bacterial outer membrane vesicles and the host–pathogen interaction. Genes Dev. 2005;19(22):2645–2655. 16291643 10.1101/gad.1299905

[21] Lécrivain A-L, Beckmann BM. Bacterial RNA in extracellular vesicles: a new regulator of host-pathogen interactions? Biochim Biophys Acta Gene Regul Mech. 2020;1863(7):194519. 32142907 10.1016/j.bbagrm.2020.194519

[22] Tsatsaronis JA, Franch-Arroyo S, Resch U, et al. Extracellular vesicle RNA: a universal mediator of microbial communication? Trends Microbiol. 2018;26(5):401–410. 29548832 10.1016/j.tim.2018.02.009

[23] Chaput N, Théry C. Exosomes: immune properties and potential clinical implementations. Semin Immunopathol. 2011;33(5):419–440. 21174094 10.1007/s00281-010-0233-9

[24] Schorey JS, Harding CV. Extracellular vesicles and infectious diseases: new complexity to an old story. J Clin Investig. 2016;126(4):1181–1189. 27035809 10.1172/JCI81132 PMC4811125

[25] Mehaffy C, Kruh-Garcia NA, Graham B, et al. Identification of *Mycobacterium tuberculosis* peptides in serum extracellular vesicles from persons with latent tuberculosis infection. J Clin Microbiol. 2020;58(6):e00393-20. 10.1128/JCM.00393-20 PMC7269374 32245831

[26] Johnstone RM, Adam M, Hammond JR, et al. Vesicle formation during reticulocyte maturation. Association of plasma membrane activities with released vesicles (exosomes). J Biol Chem. 1987;262(19):9412–9420. 3597417

[27] Witwer KW, Théry C. Extracellular vesicles or exosomes? On primacy, precision, and popularity influencing a choice of nomenclature. J Extracell Vesicles. 2019;8(1):1648167. 31489144 10.1080/20013078.2019.1648167 PMC6711079

[28] Kalluri R, LeBleu VS. The biology, function, and biomedical applications of exosomes. Science. 2020;367(6478):eaau6977. 32029601 10.1126/science.aau6977 PMC7717626

[29] Akers JC, Gonda D, Kim R, et al. Biogenesis of extracellular vesicles (EV): exosomes, microvesicles, retrovirus-like vesicles, and apoptotic bodies. J Neurooncol. 2013;113(1):1–11. 23456661 10.1007/s11060-013-1084-8 PMC5533094

[30] Schwechheimer C, Kuehn MJ. Outer-membrane vesicles from Gram-negative bacteria: biogenesis and functions. Nature Rev Microbiol. 2015;13(10):605–619. 26373371 10.1038/nrmicro3525 PMC5308417

[31] Brown L, Wolf JM, Prados-Rosales R, et al. Through the wall: extracellular vesicles in Gram-positive bacteria, mycobacteria and fungi. Nature Rev Microbiol. 2015;13(10):620–630. 26324094 10.1038/nrmicro3480 PMC4860279

[32] Briaud P, Carroll RK, Richardson AR. Extracellular vesicle biogenesis and functions in gram-positive bacteria. Infect Immun. 2020;88(12):e00433-20. 10.1128/IAI.00433-20 PMC7671900 32989035

[33] Kulp A, Kuehn MJ. Biological functions and biogenesis of secreted bacterial outer membrane vesicles. Annu Rev Microbiol. 2010;64:163–184. 20825345 10.1146/annurev.micro.091208.073413 PMC3525469

[34] Macia L, Nanan R, Hosseini-Beheshti E, et al. Host- and Microbiota-derived extracellular vesicles, immune function, and disease development. Int J Mol Sci. 2019;21(1):107. 31877909 10.3390/ijms21010107 PMC6982009

[35] Zingl FG, Kohl P, Cakar F, et al. Outer membrane vesiculation facilitates surface exchange and *in vivo* adaptation of *Vibrio cholerae*. Cell Host Microbe. 2020;27(2):225–237.e8. 31901519 10.1016/j.chom.2019.12.002 PMC7155939

[36] Caruana JC, Walper SA. Bacterial membrane vesicles as mediators of microbe – microbe and microbe – host community interactions. Front Microbiol. 2020;11:432. 32265873 10.3389/fmicb.2020.00432 PMC7105600

[37] Kim JH, Lee J, Park J, et al. Gram-negative and Gram-positive bacterial extracellular vesicles. Semin Cell Dev Biol. 2015;40:97–104. 25704309 10.1016/j.semcdb.2015.02.006

[38] Jan AT. Outer Membrane Vesicles (OMVs) of Gram-negative bacteria: a perspective update. Front Microbiol. 2017;8:1053. 28649237 10.3389/fmicb.2017.01053 PMC5465292

[39] Turnbull L, Toyofuku M, Hynen AL, et al. Explosive cell lysis as a mechanism for the biogenesis of bacterial membrane vesicles and biofilms. Nat Commun. 2016;7(1):11220. 27075392 10.1038/ncomms11220 PMC4834629

[40] Joffe LS, Nimrichter L, Rodrigues ML, et al. Potential roles of fungal extracellular vesicles during infection. mSphere. 2016;1(4):e00099-16. 10.1128/mSphere.00099-16 PMC4933989 27390779

[41] Palacios A, Coelho C, Maryam M, et al. Biogenesis and function of extracellular vesicles in gram-positive bacteria, mycobacteria, and fungi. In: Kaparakis-Liaskos M T Kufer, editors. Bacterial membrane vesicles, biogenesis, functions and applications. Cham: Springer; 2020. p. 47–74.

[42] Liu Y, Alexeeva S, Defourny KA, et al. Tiny but mighty: bacterial membrane vesicles in food biotechnological applications. Curr Opin Biotechnol. 2018;49:179–184. 28985542 10.1016/j.copbio.2017.09.001

[43] Bitencourt TA, Hatanaka O, Pessoni AM, et al. Fungal extracellular vesicles are involved in intraspecies intracellular communication. MBio. 2022;13(1):e03272-21. 10.1128/mbio.03272-21 PMC8749427 35012355

[44] Bielska E, May RC. Extracellular vesicles of human pathogenic fungi. Curr Opin Microbiol. 2019;52:90–99. 31280026 10.1016/j.mib.2019.05.007

[45] Mathieu M, Martin-Jaular L, Lavieu G, et al. Specificities of secretion and uptake of exosomes and other extracellular vesicles for cell-to-cell communication. Nat Cell Biol. 2019;21(1):9–17. 30602770 10.1038/s41556-018-0250-9

[46] Rizzo J, Rodrigues ML, Janbon G. Extracellular vesicles in fungi: past, present, and future perspectives. Front Cell Infect Microbiol. 2020;10:346. 32760680 10.3389/fcimb.2020.00346 PMC7373726

[47] Chuo STY, Chien JCY, Lai CPK. Imaging extracellular vesicles: current and emerging methods. J Biomed Sci. 2018;25(1):91. 30580764 10.1186/s12929-018-0494-5 PMC6304785

[48] Mathivanan S, Ji H, Simpson RJ. Exosomes: extracellular organelles important in intercellular communication. J Proteomics. 2010;73(10):1907–1920. 20601276 10.1016/j.jprot.2010.06.006

[49] Simons M, Raposo G. Exosomes – vesicular carriers for intercellular communication. Curr Opinion Cell Biol. 2009;21(4):575–581. 19442504 10.1016/j.ceb.2009.03.007

[50] Jadli AS, Ballasy N, Edalat P, et al. Inside(sight) of tiny communicator: exosome biogenesis, secretion, and uptake. Mol Cell Biochem. 2020;467(1–2):77–94. 32088833 10.1007/s11010-020-03703-z

[51] Record M, Subra C, Silvente-Poirot S, et al. Exosomes as intercellular signalosomes and pharmacological effectors. Biochem Pharmacol. 2011;81(10):1171–1182. 21371441 10.1016/j.bcp.2011.02.011

[52] Zhang Y, Liu Y, Liu H, et al. Exosomes: biogenesis, biologic function and clinical potential. Cell Biosci. 2019;9(1):19. 30815248 10.1186/s13578-019-0282-2 PMC6377728

[53] Cipriano MJ, Hajduk SL, Stahl P. Drivers of persistent infection: pathogen-induced extracellular vesicles. Essays Biochem. 2018;62(2):135–147. 29666211 10.1042/EBC20170083

[54] Brameyer S, Plener L, Müller A, et al. Outer membrane vesicles facilitate trafficking of the hydrophobic signaling molecule CAI-1 between *Vibrio harveyi* cells. J Bacteriol. 2018;200(15):e00740-17. 10.1128/JB.00740-17 PMC6040191 29555694

[55] Rodrigues ML, Nimrichter L, Oliveira DL, et al. Vesicular polysaccharide export in *Cryptococcus neoformans* is a eukaryotic solution to the problem of fungal trans-cell wall transport. Eukaryot Cell. 2007;6(1):48–59. 17114598 10.1128/EC.00318-06 PMC1800364

[56] Falugi F, Kim HK, Missiakas DM, et al. Role of protein a in the evasion of host adaptive immune responses by *Staphylococcus aureus*. MBio. 2013;4(5):e00575-13. 10.1128/mBio.00575-13 PMC3760252 23982075

[57] Gurung M, Moon DC, Choi CW, et al. *Staphylococcus aureus* produces membrane-derived vesicles that induce host cell death. PLoS ONE. 2011;6(11):e27958. 22114730 10.1371/journal.pone.0027958 PMC3218073

[58] Wagner T, Joshi B, Janice J, et al. *Enterococcus faecium* produces membrane vesicles containing virulence factors and antimicrobial resistance related proteins. J Proteomics. 2018;187:28–38. 29857065 10.1016/j.jprot.2018.05.017

[59] Wang X, Thompson CD, Weidenmaier C, et al. Release of *Staphylococcus aureus* extracellular vesicles and their application as a vaccine platform. Nat Commun. 2018;9(1):1379. 29643357 10.1038/s41467-018-03847-z PMC5895597

[60] Zarnowski R, Sanchez H, Covelli AS, et al. *Candida albicans* biofilm–induced vesicles confer drug resistance through matrix biogenesis. PLoS Biol. 2018;16(10):e2006872. 30296253 10.1371/journal.pbio.2006872 PMC6209495

[61] Schooling SR, Beveridge TJ. Membrane vesicles: an overlooked component of the matrices of biofilms. J Bacteriol. 2006;188(16):5945–5957. 16885463 10.1128/JB.00257-06 PMC1540058

[62] Devos S, Oudenhove LV, Stremersch S, et al. The effect of imipenem and diffusible signaling factors on the secretion of outer membrane vesicles and associated Ax21 proteins in *Stenotrophomonas maltophilia*. Front Microbiol. 2015;6:298. 25926824 10.3389/fmicb.2015.00298 PMC4396451

[63] Gill S, Catchpole R, Forterre P. Extracellular membrane vesicles in the three domains of life and beyond. FEMS Microbiol Rev. 2018;43(3):273–303. 10.1093/femsre/fuy042 PMC6524685 30476045

[64] Kuipers ME, Hokke CH, Smits HH, et al. Pathogen-derived extracellular vesicle-associated molecules that affect the host immune system: an overview. Front Microbiol. 2018;9:2182. 30258429 10.3389/fmicb.2018.02182 PMC6143655

[65] Keller MD, Ching KL, Liang F-X, et al. Decoy exosomes provide protection against bacterial toxins. Nature. 2020;579(7798):260–264. 32132711 10.1038/s41586-020-2066-6 PMC7519780

[66] Ghafourian M, Mahdavi R, Akbari Jonoush Z, et al. The implications of exosomes in pregnancy: emerging as new diagnostic markers and therapeutics targets. Cell Commun Signal. 2022;20(1):51. 35414084 10.1186/s12964-022-00853-z PMC9004059

[67] Zhang J, Li H, Fan B, et al. Extracellular vesicles in normal pregnancy and pregnancy-related diseases. J Cell Mol Med. 2020;24(8):4377–4388. 32175696 10.1111/jcmm.15144 PMC7176865

[68] Delabranche X, Berger A, Boisramé-Helms J, et al. Microparticles and infectious diseases. Médecine et maladies infectieuses. 2012;42(8):335–343. 22766273 10.1016/j.medmal.2012.05.011

[69] Janssens S, Beyaert R. Functional diversity and regulation of different interleukin-1 receptor-associated kinase (IRAK) family members. Molecular Cell. 2003;11(2):293–302. 12620219 10.1016/s1097-2765(03)00053-4

[70] Kumar H, Kawai T, Akira S. Toll-like receptors and innate immunity. Biochem Biophys Res Commun. 2009;388(4):621–625. 19686699 10.1016/j.bbrc.2009.08.062

[71] Mogensen TH. Pathogen recognition and inflammatory signaling in innate immune defenses. Clin Microbiol Rev. 2009;22(2):240–273. 19366914 10.1128/CMR.00046-08 PMC2668232

[72] Ulland TK, Ferguson PJ, Sutterwala FS. Evasion of inflammasome activation by microbial pathogens. J Clin Investig. 2015;125(2):469–477. 25642707 10.1172/JCI75254 PMC4319426

[73] Lamkanfi M, Dixit VM. Modulation of inflammasome pathways by bacterial and viral pathogens. J Immunol. 2011;187(2):597–602. 21734079 10.4049/jimmunol.1100229

[74] Admyre C, Johansson SM, Qazi KR, et al. Exosomes with immune modulatory features are present in human breast milk. J Immunol. 2007;179(3):1969–1978. 17641064 10.4049/jimmunol.179.3.1969

[75] Bhatnagar S, Shinagawa K, Castellino FJ, et al. Exosomes released from macrophages infected with intracellular pathogens stimulate a proinflammatory response *in vitro* and *in vivo*. Blood. 2007;110(9):3234–3244. 17666571 10.1182/blood-2007-03-079152 PMC2200902

[76] Schorey JS, Bhatnagar S. Exosome function: from tumor immunology to pathogen biology. Traffic. 2008;9(6):871–881. 18331451 10.1111/j.1600-0854.2008.00734.x PMC3636814

[77] Smyth LA, Ratnasothy K, Tsang JYS, et al. CD73 expression on extracellular vesicles derived from CD4+CD25+Foxp3+ T cells contributes to their regulatory function. Eur J Immunol. 2013;43(9):2430–2440. 23749427 10.1002/eji.201242909

[78] Zhang H, Xie Y, Li W, et al. CD4+ T cell-released exosomes inhibit CD8+ cytotoxic T-lymphocyte responses and antitumor immunity. Cell Mol Immunol. 2011;8(1):23–30. 21200381 10.1038/cmi.2010.59 PMC4002994

[79] Finlay BB, McFadden G. Anti-immunology: evasion of the host immune system by bacterial and viral pathogens. Cell. 2006;124(4):767–782. 16497587 10.1016/j.cell.2006.01.034

[80] Iwasaki A, Medzhitov R. Regulation of adaptive immunity by the innate immune system. Science. 2010;327(5963):291–295. 20075244 10.1126/science.1183021 PMC3645875

[81] Li D, Long Y, Wang T, et al. Epidemiology of Hepatitis C virus infection in highly endemic HBV areas in China. PLoS ONE. 2013;8(1):e54815. 23372775 10.1371/journal.pone.0054815 PMC3555996

[82] Pleet ML, Erickson J, DeMarino C, et al. Ebola virus VP40 modulates cell cycle and biogenesis of extracellular vesicles. J Infect Dis. 2018;218(suppl_5):S365–87. 30169850 10.1093/infdis/jiy472 PMC6249571

[83] Editorial. Biomarkers on a roll. Nature Biotechnol. 2010;28(5):431. 20458308 10.1038/nbt0510-431

[84] Kapasi AJ, Dittrich S, González IJ, et al. Host biomarkers for distinguishing bacterial from non-bacterial causes of acute febrile illness: a comprehensive review. PLoS One. 2016;11(8):e0160278. 27486746 10.1371/journal.pone.0160278 PMC4972355

[85] Lee H. Procalcitonin as a biomarker of infectious diseases. Korean J Internal Medi. 2013;28(3):285–291. 10.3904/kjim.2013.28.3.285 PMC3654123 23682219

[86] Havelka A, Sejersen K, Venge P, et al. Calprotectin, a new biomarker for diagnosis of acute respiratory infections. Sci Rep. 2020;10(1):4208. 32144345 10.1038/s41598-020-61094-z PMC7060262

[87] Lubell Y, Althaus T. Biomarker tests for bacterial infection—a costly wait for the holy grail. Lancet Infect Dis. 2017;17(4):369–370. 28346177 10.1016/S1473-3099(17)30124-X

[88] Vijayan AL, Vanimaya, Ravindran S, et al. Procalcitonin: a promising diagnostic marker for sepsis and antibiotic therapy. J Intensive Care. 2017;5(1):51. 28794881 10.1186/s40560-017-0246-8 PMC5543591

[89] Andaloussi SE, Mäger I, Breakefield XO, et al. Extracellular vesicles: biology and emerging therapeutic opportunities. Nat Rev Drug Discov. 2013;12(5):347–357. 23584393 10.1038/nrd3978

[90] Fuhrmann G, Neuer AL, Herrmann IK. Extracellular vesicles – a promising avenue for the detection and treatment of infectious diseases? Eur J Pharm Biopharm. 2017;118:56–61. 28396279 10.1016/j.ejpb.2017.04.005

[91] Hosseini‐beheshti E, Grau GER. Extracellular vesicles as mediators of immunopathology in infectious diseases. Immunol Cell Biol. 2018;96(7):694–703. 10.1111/imcb.12044 29577413

[92] Thompson AG, Gray E, Heman-Ackah SM, et al. Extracellular vesicles in neurodegenerative disease — pathogenesis to biomarkers. Nat Rev Neurol. 2016;12(6):346–357. 27174238 10.1038/nrneurol.2016.68

[93] Kang H, Kim J, Park J. Methods to isolate extracellular vesicles for diagnosis. Micro Nano Syst Lett. 2017;5(1):15.

[94] Shao H, Im H, Castro CM, et al. New technologies for analysis of extracellular vesicles. Chem Rev. 2018;118(4):1917–1950. 29384376 10.1021/acs.chemrev.7b00534 PMC6029891

[95] Tulkens J, Wever OD, Hendrix A. Analyzing bacterial extracellular vesicles in human body fluids by orthogonal biophysical separation and biochemical characterization. Nat Protoc. 2020;15(1):40–67. 31776460 10.1038/s41596-019-0236-5

[96] Badi SA, Bruno SP, Moshiri A, et al. Small RNAs in outer membrane vesicles and their function in host-microbe interactions. Front Microbiol. 2020;11:1209. 32670219 10.3389/fmicb.2020.01209 PMC7327240

[97] Vitse J, Devreese B. The contribution of membrane vesicles to bacterial pathogenicity in cystic fibrosis infections and healthcare associated pneumonia. Front Microbiol. 2020;11:630. 32328052 10.3389/fmicb.2020.00630 PMC7160670

[98] Nadeem A, Oscarsson J, Wai SN. Delivery of virulence factors by bacterial membrane vesicles to mammalian host cells. In: Kaparakis-Liaskos M T Kufer, editors. Bacterial membrane vesicles, biogenesis, functions and applications. Cham: Springer; 2020. p. 131–158.

[99] Armistead B, Quach P, Snyder JM, et al. Hemolytic membrane vesicles of group B *Streptococcus* promote infection. J Infect Dis. 2021;223(8):1488–1496. 32861213 10.1093/infdis/jiaa548 PMC8064051

[100] Liu GY, Nizet V. Color me bad: microbial pigments as virulence factors. Trends Microbiol. 2009;17(9):406–413. 19726196 10.1016/j.tim.2009.06.006 PMC2743764

[101] Los FCO, Randis TM, Aroian RV, et al. Role of pore-forming toxins in bacterial infectious diseases. Microbiol Mol Biol Rev. 2013;77(2):173–207. 23699254 10.1128/MMBR.00052-12 PMC3668673

[102] Bozhokina E, Kever L, Khaitlina S. The *Serratia grimesii* outer membrane vesicles‐associated grimelysin triggers bacterial invasion of eukaryotic cells. Cell Biol Int. 2020;44(11):2275–2283. 32749752 10.1002/cbin.11435

[103] World Health Organization. Tuberculosis [Internet]. 2021. Available from: https://www.who.int/news-room/fact-sheets/detail/tuberculosis

[104] Tufariello JM, Chan J, Flynn JL. Latent tuberculosis: mechanisms of host and bacillus that contribute to persistent infection. Lancet Infect Dis. 2003;3(9):578–590. 12954564 10.1016/s1473-3099(03)00741-2

[105] Mirzaei R, Babakhani S, Ajorloo P, et al. The emerging role of exosomal miRnas as a diagnostic and therapeutic biomarker in *Mycobacterium tuberculosis* infection. Mol Med. 2021;27(1):34. 33794771 10.1186/s10020-021-00296-1 PMC8017856

[106] Mori M, Pieters J. Getting in and staying alive: role for coronin 1 in the survival of pathogenic mycobacteria and naïve T cells. Front Immunol. 2018;9:1592. 30042765 10.3389/fimmu.2018.01592 PMC6049072

[107] Layre E. Trafficking of *Mycobacterium tuberculosis* envelope components and release within extracellular vesicles: host-pathogen interactions beyond the wall. Front Immunol. 2020;11:1230. 32765485 10.3389/fimmu.2020.01230 PMC7378356

[108] Chai Q, Wang L, Liu CH, et al. New insights into the evasion of host innate immunity by *Mycobacterium tuberculosis*. Cell Mol Immunol. 2020;17(9):901–913. 32728204 10.1038/s41423-020-0502-z PMC7608469

[109] Miranda MS, Breiman A, Allain S, et al. The tuberculous granuloma: an unsuccessful host defence mechanism providing a safety shelter for the bacteria? Clin Dev Immunol. 2012;2012:139127. 22811737 10.1155/2012/139127 PMC3395138

[110] Pagán AJ, Yang C-T, Cameron J, et al. Myeloid growth factors promote resistance to mycobacterial infection by curtailing granuloma necrosis through macrophage replenishment. Cell Host Microbe. 2015;18(1):15–26. 26159717 10.1016/j.chom.2015.06.008 PMC4509513

[111] Liu L, Zhai K, Chen Y, et al. Effect and mechanism of *Mycobacterium tuberculosis* lipoprotein LpqH in NLRP3 inflammasome activation in mouse Ana-1 macrophage. In: Ashwood P, editor. BioMed research international. Vol. 2021. 2021. p. 1–8. DOI:10.1155/2021/8239135 PMC7803426 33490276

[112] Palacios A, Sampedro L, Sevilla IA, et al. *Mycobacterium tuberculosis* extracellular vesicle-associated lipoprotein LpqH as a potential biomarker to distinguish paratuberculosis infection or vaccination from tuberculosis infection. BMC Vet Res. 2019;15(1):188. 31174546 10.1186/s12917-019-1941-6 PMC6555097

[113] Harding CV, Boom WH. Regulation of antigen presentation by *Mycobacterium tuberculosis*: a role for Toll-like receptors. Nature Rev Microbiol. 2010;8(4):296–307. 20234378 10.1038/nrmicro2321 PMC3037727

[114] Wong D, Bach H, Sun J, et al. *Mycobacterium tuberculosis* protein tyrosine phosphatase (PtpA) excludes host vacuolar-H+–ATPase to inhibit phagosome acidification. Proc Nat Acad Sci. 2011;108(48):19371–19376. 22087003 10.1073/pnas.1109201108 PMC3228452

[115] Prados-Rosales R, Weinrick BC, Piqué DG, et al. Role for *Mycobacterium tuberculosis* membrane vesicles in iron acquisition. J Bacteriol. 2014;196(6):1250–1256. 24415729 10.1128/JB.01090-13 PMC3957709

[116] Pan S-J, Tapley A, Adamson J, et al. Biomarkers for tuberculosis based on secreted, species-specific, bacterial small molecules. J Infect Dis. 2015;212(11):1827–1834. 26014799 10.1093/infdis/jiv312 PMC4633767

[117] Kleinnijenhuis J, Oosting M, Joosten LAB, et al. Innate immune recognition of *Mycobacterium tuberculosis*. Clin Dev Immunol. 2011;2011:405310. 21603213 10.1155/2011/405310 PMC3095423

[118] Sun Y-F, Pi J, Xu J-F. Emerging role of exosomes in tuberculosis: from immunity regulations to vaccine and immunotherapy. Front Immunol. 2021;12:628973. 33868247 10.3389/fimmu.2021.628973 PMC8047325

[119] Sande OJ, Karim AF, Li Q, et al. Mannose-capped lipoarabinomannan from *Mycobacterium tuberculosis* induces CD4+ T cell anergy via GRAIL. J Immunol. 2016;196(2):691–702. 26667170 10.4049/jimmunol.1500710 PMC4707121

[120] Correia-Neves M, Fröberg G, Korshun L, et al. Biomarkers for tuberculosis: the case for lipoarabinomannan. ERJ Open Res. 2019;5(1):00115–2018. 30775376 10.1183/23120541.00115-2018 PMC6368998

[121] Richardson ET, Shukla S, Sweet DR, et al. Toll-like receptor 2-dependent extracellular signal-regulated kinase signaling in *Mycobacterium tuberculosis*-infected macrophages drives anti-inflammatory responses and inhibits Th1 polarization of responding T cells. Infect Immun. 2015;83(6):2242–2254. 25776754 10.1128/IAI.00135-15 PMC4432743

[122] Fernández‐Messina L, Gutiérrez‐Vázquez C, Rivas‐García E, et al. Immunomodulatory role of microRnas transferred by extracellular vesicles. Biol Cell. 2015;107(3):61–77. 25564937 10.1111/boc.201400081 PMC5010100

[123] Lyu L, Zhang X, Li C, et al. Small RNA profiles of serum exosomes derived from individuals with latent and active tuberculosis. Front Microbiol. 2019;10:1174. 31191492 10.3389/fmicb.2019.01174 PMC6546874

[124] Moore DAJ, Evans CAW, Gilman RH, et al. Microscopic-observation drug-susceptibility assay for the diagnosis of TB. N Engl J Med. 2006;355(15):1539–1550. 17035648 10.1056/NEJMoa055524 PMC1780278

[125] Gill CM, Dolan L, Piggott LM, et al. New developments in tuberculosis diagnosis and treatment. Breathe. 2022;18:210149. 35284018 10.1183/20734735.0149-2021 PMC8908854

[126] Acharya B, Acharya A, Gautam S, et al. Advances in diagnosis of Tuberculosis: an update into molecular diagnosis of *Mycobacterium tuberculosis*. Mol Biol Rep. 2020;47(5):4065–4075. 32248381 10.1007/s11033-020-05413-7

[127] Dahiya B, Khan A, Mor P, et al. Detection of *Mycobacterium tuberculosis* lipoarabinomannan and CFP-10 (Rv3874) from urinary extracellular vesicles of tuberculosis patients by immuno-PCR. Pathog Dis. 2019;77(5):77. 10.1093/femspd/ftz049 31549171

[128] Cilloniz C, Martin-Loeches I, Garcia-Vidal C, et al. Microbial etiology of pneumonia: epidemiology, diagnosis and resistance patterns. Int J Mol Sci. 2016;17(12):2120. 27999274 10.3390/ijms17122120 PMC5187920

[129] Olaya-Abril A, Prados-Rosales R, McConnell MJ, et al. Characterization of protective extracellular membrane-derived vesicles produced by *Streptococcus pneumoniae*. J Proteomics. 2014;106:46–60. 24769240 10.1016/j.jprot.2014.04.023

[130] Prayle A, Atkinson M, Smyth A. Pneumonia in the developed world. Paediatr Respir Rev. 2011;12(1):60–69. 21172677 10.1016/j.prrv.2010.09.012

[131] Yerneni SS, Werner S, Azambuja JH, et al. Bacterial extracellular vesicle mediated host-pathogen interactions in pneumococcal infections. J Immunol Res 2020;9:e00559-18.

[132] Behrens F, Funk-Hilsdorf TC, Kuebler WM, et al. Bacterial membrane vesicles in pneumonia: from mediators of virulence to innovative vaccine candidates. Int J Mol Sci. 2021;22(8):3858. 33917862 10.3390/ijms22083858 PMC8068278

[133] Munford RS, Hall CL, Lipton JM, et al. Biological activity, Lipoprotein-binding behavior, and *in vivo* disposition of extracted and native forms of *Salmonella typhimurium* lipopolysaccharides. J Clin Investig. 1982;70(4):877–888. 6749904 10.1172/JCI110684 PMC370296

[134] Bauman SJ, Kuehn MJ. Purification of outer membrane vesicles from *Pseudomonas aeruginosa* and their activation of an IL-8 response. Microbes Infect. 2006;8(9–10):2400–2408. 16807039 10.1016/j.micinf.2006.05.001 PMC3525494

[135] Renelli M, Matias V, Lo RY, et al. DNA-containing membrane vesicles of *Pseudomonas aeruginosa* PAO1 and their genetic transformation potential. Microbiology. 2004;150(7):2161–2169. 15256559 10.1099/mic.0.26841-0

[136] Lee JC, Lee EJ, Lee JH, et al. *Klebsiella pneumoniae* secretes outer membrane vesicles that induce the innate immune response. FEMS Microbiol Lett. 2012;331(1):17–24. 22428779 10.1111/j.1574-6968.2012.02549.x

[137] Codemo M, Muschiol S, Iovino F, et al. Immunomodulatory effects of pneumococcal extracellular vesicles on cellular and humoral host defenses. MBio. 2018;9(2):e00559-18. 10.1128/mBio.00559-18 PMC5893880 29636428

[138] Jhelum H, Sori H, Sehgal D. A novel extracellular vesicle-associated endodeoxyribonuclease helps *Streptococcus pneumoniae* evade neutrophil extracellular traps and is required for full virulence. Sci Rep. 2018;8(1):7985. 29789571 10.1038/s41598-018-25865-z PMC5964101

[139] Lanyu Z, Feilong H. Emerging role of extracellular vesicles in lung injury and inflammation. Biomed Pharmacother. 2019;113:108748. 30877881 10.1016/j.biopha.2019.108748

[140] Lee H, Zhang D, Laskin DL, et al. Functional evidence of pulmonary extracellular vesicles in infectious and noninfectious lung inflammation. J Immunol. 2018;201(5):1500–1509. 29997122 10.4049/jimmunol.1800264 PMC6109965

[141] Lee H, Groot M, Pinilla-Vera M, et al. Identification of miRNA-rich vesicles in bronchoalveolar lavage fluid: insights into the function and heterogeneity of extracellular vesicles. J Control Release. 2019;294:43–52. 30529727 10.1016/j.jconrel.2018.12.008 PMC6372374

[142] Murdoch DR, O’brien KL, Driscoll AJ, et al. Laboratory methods for determining pneumonia etiology in children. Clinl Infect Dis. 2012;54(suppl_2):S146–52. 10.1093/cid/cir1073 22403229

[143] Douglas IS. New diagnostic methods for pneumonia in the ICU. Curr Opin Infect Dis. 2016;29(2):197–204. 26859725 10.1097/QCO.0000000000000249

[144] Bakare OO, Keyster M, Pretorius A. Identification of biomarkers for the accurate and sensitive diagnosis of three bacterial pneumonia pathogens using *in silico* approaches. BMC Mol Cell Biol. 2020;21(1):82. 33218302 10.1186/s12860-020-00328-4 PMC7678116

[145] Piszczatowska K, Czerwaty K, Cyran AM, et al. The emerging role of small extracellular vesicles in inflammatory airway diseases. Diagnostics. 2021;11(2):222. 33540806 10.3390/diagnostics11020222 PMC7913078

[146] Huang F, Bai J, Zhang J, et al. Identification of potential diagnostic biomarkers for pneumonia caused by adenovirus infection in children by screening serum exosomal microRnas. Mol Med Rep. 2019;19:4306–4314. 30942467 10.3892/mmr.2019.10107 PMC6471624

[147] Sun Y, Xian Y, Duan Z, et al. Diagnostic potential of microRnas in extracellular vesicles derived from bronchoalveolar lavage fluid for pneumonia—a preliminary report. Cells. 2022;11(19):2961. 36230923 10.3390/cells11192961 PMC9564323

[148] Jung AL, Jørgensen MM, Bæk R, et al. Surface proteome of plasma extracellular vesicles as biomarkers for pneumonia and acute exacerbation of chronic obstructive pulmonary disease. J Infect Dis. 2019;221:325–335. 10.1093/infdis/jiz460 31617573

[149] Hsu C-W, Suk C-W, Hsu Y-P, et al. Sphingosine-1-phosphate and CRP as potential combination biomarkers in discrimination of COPD with community-acquired pneumonia and acute exacerbation of COPD. Respir Res. 2022;23(1):63. 35307030 10.1186/s12931-022-01991-1 PMC8935698

[150] Jung AL, Schmeck B, Wiegand M, et al. The clinical role of host and bacterial-derived extracellular vesicles in pneumonia. Adv Drug Delivery Rev. 2021;176:113811. 10.1016/j.addr.2021.05.021 34022269

[151] Hwang W, Shimizu M, Lee J-W. Role of extracellular vesicles in severe pneumonia and sepsis. Expert Opin Biol Ther. 2022;22(6):747–762. 35418256 10.1080/14712598.2022.2066470 PMC9971738

[152] Braunsdorf C, Mailänder-Sánchez D, Schaller M. Fungal sensing of host environment: fungal sensing. Cell Microbiol. 2016;18(9):1188–1200. 27155351 10.1111/cmi.12610

[153] Romani L. Immunity to fungal infections. Nat Rev Immunol. 2004;4(1):11–24. 10.1038/nri1255 14661066

[154] Albuquerque PC, Nakayasu ES, Rodrigues ML, et al. Vesicular transport in *Histoplasma capsulatum*: an effective mechanism for trans-cell wall transfer of proteins and lipids in ascomycetes. Cell Microbiol. 2008;10(8):1695–1710. 18419773 10.1111/j.1462-5822.2008.01160.x PMC2562661

[155] Leone F, Bellani L, Muccifora S, et al. Analysis of extracellular vesicles produced in the biofilm by the dimorphic yeast *Pichia fermentans*. J Cell Physiol. 2018;233(4):2759–2767. 28256706 10.1002/jcp.25885

[156] Vargas G, Rocha JDB, Oliveira DL, et al. Compositional and immunobiological analyses of extracellular vesicles released by *Candida albicans*. Cell Microbiol. 2015;17(3):389–407. 25287304 10.1111/cmi.12374

[157] Ellerbroek PM, Lefeber DJ, van Veghel R, et al. O-Acetylation of Cryptococcal capsular glucuronoxylomannan is essential for interference with neutrophil migration. J Immunol. 2004;173(12):7513–7520. 15585878 10.4049/jimmunol.173.12.7513

[158] Rodrigues ML, Nakayasu ES, Oliveira DL, et al. Extracellular vesicles produced by *Cryptococcus neoformans* contain protein components associated with virulence. Eukaryot Cell. 2008;7(1):58–67. 18039940 10.1128/EC.00370-07 PMC2224146

[159] Oliveira DL, Freire-de-Lima CG, Nosanchuk JD, et al. Extracellular vesicles from *Cryptococcus neoformans* modulate macrophage functions. Infect Immun. 2010;78(4):1601–1609. 20145096 10.1128/IAI.01171-09 PMC2849392

[160] Panepinto J, Komperda K, Frases S, et al. Sec6‐dependent sorting of fungal extracellular exosomes and laccase of *Cryptococcus neoformans*. Mol Microbiol. 2009;71(5):1165–1176. 19210702 10.1111/j.1365-2958.2008.06588.x

[161] Eisenman HC, Frases S, Nicola AM, et al. Vesicle-associated melanization in *Cryptococcus neoformans*. Microbiology. 2009;155(12):3860–3867. 19729402 10.1099/mic.0.032854-0 PMC2889422

[162] Liu D, Wei L, Guo T, et al. Detection of DOPA-melanin in the dimorphic fungal pathogen *Penicillium marneffei* and its effect on macrophage phagocytosis *in vitro*. PLoS ONE. 2014;9(3):e92610. 24647795 10.1371/journal.pone.0092610 PMC3960263

[163] Bielska E, Sisquella MA, Aldeieg M, et al. Pathogen-derived extracellular vesicles mediate virulence in the fatal human pathogen *Cryptococcus gattii*. Nat Commun. 2018;9(1):1556. 29674675 10.1038/s41467-018-03991-6 PMC5908794

[164] Gil-Bona A, Monteoliva L, Gil C. Global proteomic profiling of the secretome of *Candida albicans* ecm33 cell wall mutant reveals the involvement of ecm33 in sap2 secretion. J Proteome Res. 2015;14(10):4270–4281. 26290404 10.1021/acs.jproteome.5b00411

[165] Freitas D, Balmaña M, Poças J, et al. Different isolation approaches lead to diverse glycosylated extracellular vesicle populations. J Extracell Vesicles. 2019;8(1):1621131. 31236201 10.1080/20013078.2019.1621131 PMC6571546

[166] Halder LD, Eah J, Hasan MZ, et al. Immune modulation by complement receptor 3-dependent human monocyte TGF-β1-transporting vesicles. Nat Commun. 2020;11(1):2331. 32393780 10.1038/s41467-020-16241-5 PMC7214408

[167] Kabir V, Maertens J, Kuypers D. Fungal infections in solid organ transplantation: an update on diagnosis and treatment. Transplantation Rev. 2019;33(2):77–86. 10.1016/j.trre.2018.12.001 30579665

[168] Guarner J, Brandt ME. Histopathologic diagnosis of fungal infections in the 21st Century. Clinical Microbiology Reviews. 2011;24(2):247–280. 21482725 10.1128/CMR.00053-10 PMC3122495

[169] Lass-Flörl C. Current challenges in the diagnosis of fungal infections. Methods Mol Biol. 2017;1508:3–15. 27837496 10.1007/978-1-4939-6515-1_1

[170] Gomez CA, Budvytiene I, Zemek AJ, et al. Performance of targeted fungal sequencing for culture-independent diagnosis of invasive fungal disease. Clinl Infect Dis. 2017;65(12):2035–2041. 10.1093/cid/cix728 29020284

[171] Vaz C, Pitarch A, Gómez-Molero E, et al. Mass spectrometry-based proteomic and immunoproteomic analyses of the *Candida albicans* hyphal secretome reveal diagnostic biomarker candidates for invasive candidiasis. J Fungi. 2021;7(7):501. 10.3390/jof7070501 PMC8306665 34201883

[172] Koo S, Thomas HR, Daniels SD, et al. A breath fungal secondary metabolite signature to diagnose invasive aspergillosis. Clinl Infect Dis. 2014;59(12):1733–1740. 10.1093/cid/ciu725 PMC4311178 25342502

[173] Herkert PF, Amatuzzi RF, Alves LR, et al. Extracellular vesicles as vehicles for the delivery of biologically active fungal molecules. Curr Protein Pept Sci. 2019;20(10):1027–1036. 31142247 10.2174/1389203720666190529124055

[174] Martínez-López R, Hernáez ML, Redondo E, et al. *Candida albicans* hyphal extracellular vesicles are different from yeast ones, carrying an active proteasome complex and showing a different role in host immune response. Microbiol Spectr. 2022;10(3):e00698-22. 10.1128/spectrum.00698-22 PMC9241596 35604172

[175] Zamith-Miranda D, da Silva RP, Couvillion SP, et al. Omics approaches for understanding biogenesis, composition and functions of fungal extracellular vesicles. Front Genet. 2021;12:648524. 34012462 10.3389/fgene.2021.648524 PMC8126698

